# Biologic and synthetic skin substitutes: An overview

**DOI:** 10.4103/0970-0358.70712

**Published:** 2010-09

**Authors:** Ahmad Sukari Halim, Teng Lye Khoo, Shah Jumaat Mohd. Yussof

**Affiliations:** Reconstructive Sciences Unit, School of Medical Sciences, Universiti Sains Malaysia, 16150 Kubang Kerian, Kelantan, Malaysia

**Keywords:** Skin substitute, burn, biological dressing

## Abstract

The current trend of burn wound care has shifted to more holistic approach of improvement in the long-term form and function of the healed burn wounds and quality of life. This has demanded the emergence of various skin substitutes in the management of acute burn injury as well as post burn reconstructions. Skin substitutes have important roles in the treatment of deep dermal and full thickness wounds of various aetiologies. At present, there is no ideal substitute in the market. Skin substitutes can be divided into two main classes, namely, biological and synthetic substitutes. The biological skin substitutes have a more intact extracellular matrix structure, while the synthetic skin substitutes can be synthesised on demand and can be modulated for specific purposes. Each class has its advantages and disadvantages. The biological skin substitutes may allow the construction of a more natural new dermis and allow excellent re-epithelialisation characteristics due to the presence of a basement membrane. Synthetic skin substitutes demonstrate the advantages of increase control over scaffold composition. The ultimate goal is to achieve an ideal skin substitute that provides an effective and scar-free wound healing.

## INTRODUCTION

The current trend of burn wound care has been shifted from merely achieving satisfactory survival rate to improvement in the long-term form and function of the healed burn wounds and quality of life. The change in the trend has demanded the emergence of various skin substitutes in the management of burn injury.

The timely restoration of skin protective functions is the key to the successful treatment of burn victims with various severity of damaged skin. Conventionally, autologous split or full-thickness skin graft have been recognised as the best definitive burn wound coverage, but it is constrained by the limited available sources, especially in major burns. Donor site morbidities in term of additional wounds and scarring are also of concern of the autograft application.

Skin substitutes are required in the acutely burned patients as well as in those requiring extensive post burn reconstructions. These have important impact in the care and outcome in the burn victims.

## DEFINITION

Skin substitutes are heterogeneous group of wound coverage materials that aid in would closure and replace the functions of the skin, either temporarily or permanently, depending on the product characteristics. These substances are alternatives to the standard wound coverage in circumstances when standard therapies are not desirable.[1]

## HISTORY

The history of application of skin substitutes dates back to the first written report on the skin xenograft in the 15th century BC as mentioned in the Papyrus of Ebers. The clinical use of human skin allograft was first described in the manuscript of Branca of Sicily in 1503. Various skin substitutes that were tested over time, such as human skin allograft, xenograft and amnion, are still being used at various burn centres all over the world. With the advent and progress of biotechnology and tissue engineering, a wide array of skin substitutes is now available in the market for the treatment of burn injury.

## IMPORTANT FACTORS TO BE CONSIDERED

There are several important factors that are taken into consideration in the decision to use the skin substitutes in burn wound management. These include the depth of burn, availability of donor site, likelihood of wound infection, sites of burn, likelihood of contracture, aesthetic outcome, relative cost, time consumption and experience of the burn surgeons.[2]

The skin substitutes provide rapid wound coverage solution that may require less vascularised wound bed, increase in the dermal component of healed wound, reduce or removed inhibitory factors of wound healing, reduced inflammatory response and subsequent scarring.[1] However, these skin substitutes generally necessitate higher cost, expertise and experience.

## WHAT IS THE IDEAL BURN WOUND COVERAGE?

The optimal skin substitute will provide for immediate replacement of both the lost dermis and epidermis, with permanent wound coverage.[3] Other features of the ideal skin substitute should have the following features:[1]

- Able to resist infection
- Able to prevent water loss
- Able to withstand the shear forces
- Cost effective
- Widely available
- Long shelf life and easy to store
- Lack of antigenicity
- Flexible in thickness
- Durable with long-term wound stability
- Can be conformed to irregular wound surfaces and
- Easy to be secured and applied

To date, there is no ideal skin substitute available that fulfills all the above-mentioned features. Currently, tissue engineering and biotechnology are gearing towards the direction of creating an optimal skin substitute.

## CLASSIFICATIONS

There are various ways to classify the skin substitutes. A classification was proposed based on composition as follows:[4]

Class I: Temporary impervious dressing materials

a) single layer materials

- naturally occurring or biological dressing substitute, e.g. amniotic membrane, potato peel
- synthetic dressing substitute, e.g. synthetic polymer sheet (Tegaderm®, Opsite®), polymer foam or spray

b) bi-layered tissue engineered materials, e.g. TransCyte®

Class II: Single layer durable skin substitutes

- Epidermal substitutes, e.g. cultured epithelial autograft (CEA), Apligraft®
- Dermal substuitutes

- bovine collagen sheet, e.g. Kollagen®
- porcine collagen sheet
- bovine dermal matrix, e.g. Matriderm®
- human dermal matrix, e.g. Alloderm®

Class III: Composite skin substitutes

a) Skin graft

- Allograft
- Xenograft

b) Tissue engineered skin

- Dermal regeneration template, e.g. Integra®
- Biobrane®

From the practical point of view, the skin substitutes are best classified as temporary or permanent and synthetic or biological.

### Temporary skin substitutes

Temporary skin substitutes provide transient physiologic wound closure, including protection from mechanical trauma, physical barrier to bacteria and creation of a moist wound environment.[3]

The common uses for temporary skin substitutes include:

1. for dressing on donor sites to facilitate epithelialisation and pain control,
2. for dressing on clean superficial wounds until epithelialisation
3. to provide temporary physiological closure of deep dermal and full thickness wounds after excision while awaiting autografting,
4. as sandwich graft technique over the widely meshed autografts and
5. as a “test” graft in questionable wound beds.

### Permanent skin substitutes

The permanent skin substitutes have the roles of permanently achieving wound closure, replacing the skin components and providing a higher quality skin replacement than the thin autogolous skin graft.

### Biological skin substitutes

These skin substitutes which act temporarily like skin, have the advantages of being relatively abundant in supply and not expensive. While the synthetic skin substitutes can be synthesised on demand and can be modulated for specific purposes with increased control over scaffold composition, the biological skin substitutes have a more intact and native ECM structure which may allow the construction of a more natural new dermis. They also allow excellent re-epithelialisation characteristics due to the presence of a basement membrane. However, natural constructs can exhibit problems with slow vascularisation of the material. The most widely used biological substitute worldwide are cadaveric skin allograft, porcine skin xenograft and amnion.

### Xenograft

Xenografts are skin substitutes harvested from the animals for use as temporary graft in human. The earliest reported xenograft application for wound coverage was as early as 1500 BC with the use of frog skin. Porcine skin allograft is the most commonly used xenograft in modern practice of burn care.

Xenografts are indicated for clean partial-thickness burns as temporary coverage. The recent modifications to the porcine skin include aldehyde cross-linking and silver ion impregnation to increase the antimicrobial properties.[1]

### Skin allograft

The use skin allograft dates back to the World War II. The cadaveric skin allograft application is one of the most commonly applied skin substitutes in burn wound management in many major burn centres all over the world. There are reports on skin allograft from living donors. Depending on the methods of processing and storage, there are two main types of cadaveric skin allografts, cryopreserved allograft and glycerol-preserved allograft (GPA). The GPA is more popular and commonly used in clinical practice.[5]

The skin allograft has been indicated for wound bed preparation, definitive dressing and sandwich grafting technique. It is also used as an interim coverage after burn scar release. Our report on the experience and outcome of application of GPA in series of 43 consecutive cases with mean 28.7% total body surface area of burns has concluded the versatility of GPA in burn wounds of various depth. The GPA has adhered to the wound for an average of 8.4 days before rejection. The autograft take rate of 88.4% after wound bed preparation with GPA was achieved. The autograft take rate was 74.4% when GPA was used for sandwich grafting technique. The GPA is applied to cover the partial-thickness burn as a definitive biological dressing until the underlying burn wounds have epithelialised. It is noted that GPA allows painless and easy dressing changes, which is particularly important and beneficial in paediatric patients with burns in order to avoid the physical and psychological stress.[5]

A viable skin allograft can re-vascularise by inosculation like autologous split skin graft. In addition, skin allograft can provide growth factors and essential cytokines while creating chemotaxis and proliferation at the wound beds. As the skin allograft can increase vascularity in the wound bed, including promoting angiogenesis with enhanced capillary ingrowth on the wound bed, it has been used for burn wound bed preparation. The freshly excised burn wound could be optimised and conditioned to prepare for subsequent autografting by application of skin allograft.

When the skin allograft is used for sandwich grafting technique to overlay the autograft, the allograft prevents desiccation of the wound bed in the interstices of widely expanded autografts and also reduces bacterial colonisation. The autograft is protected from shear. The epithelialisation of the wound bed is also accelerated when skin allograft is applied.

### Amnion

The amnion is a thin semi-transparent tissue forming the innermost layer of the foetal membrane. The amnion has been used as biological dressings for burns since 1910. As fresh amnion carries risk contaminations and disease transmission, amnion is collected from placentae of selected and screened donors. Various preservation methods have been introduced, including cyopreservation in liquid nitrogen, preservation in silver nitrate, storage in antibiotics solution, glycerol-preserved sheets, dried sheets and gamma-irradiated sheets.[6]

It has been claimed to be one of the most effective biological skin substitutes used in burn wounds, with efficiency of maintaining low bacteria count. It also has advantages of reducing loss of protein, electrolytes and fluids, decreasing the risk infection, minimising pain, acceleration of wound healing and good handling properties.

Amnion is primarily used for covering partial-thickness burns until complete healing. It is particularly useful for superficial partial-thickness facial burns.[6] When used in facial burns, it is noted to be adhesive, conformable and easily removable. It is also used for temporary coverage in wound bed preparation and sandwich grafting technique.

### Cultured epithelial autografts

The culture of keratinocytes is an important advance in the burn care. CEA was first reported in the clinical use in 1981 in extensive full thickness burns. A large surface area of keratinocytes can be obtained from the relatively small biopsy of healthy skin from the patient. The autologous keratinocytes are isolated, cultured and expanded into sheets over periods of 3–5 weeks. The technique of suspension in fibrin glue has reduced the time for clinical use to 2 weeks.[7]

CEA avoids the mesh aspect of split skin autografts and discomfort of donor site after skin harvesting. It is however limited by the fragility and difficulty of handling, unpredictable take rate and high cost.

### Synthetic skin substitutes

Synthetic skin substitutes are constructed out of non-biological molecules and polymers that are not present in normal skin.[8] These constructs should be stable, biodegradable and provide an adequate environment for the regeneration of tissue. It should maintain its three-dimensional structure for at least 3 weeks to allow ingrowths of blood vessels, fibroblast and coverage by epithelial cells. Biodegradation should preferably take place after this period. This process should occur without massive foreign body reaction as this process would increase the inflammatory response, which may be associated with profound scarring. It should also be composed of immunocompatible materials to avoid immunoreactive processes.

The artificial nature of these skin substitutes has some distinct advantages and disadvantages when compared to natural biological structures. The composition and properties of the product can be much more precisely controlled. Various additives such as growth factors and matrix components can be added to enhance the effect. These products could also avoid complications due to potential disease transmission. However, these synthetic skin substitutes generally lack basement membrane and their architecture do not resemble native skin. The use of non-biological components can be problematic when trying to produce a biologically compatible material.[8]

There are several synthetic skin substitutes that are available for wound coverage. However, there are also substantial number of synthetic substitutes undergoing *in vitro* or animal testing.[9–11] Amongst the synthetic skin substitutes available in the market are Biobrane®, Dermagraft®, Integra®, Apligraft®, Matriderm®, Orcel®, Hyalomatrix® and Renoskin®.

### Biobrane®

Biobrane® (Dow-Hickham, Sugarland, TX, USA) consists of an inner layer of nylon mesh that allows fibrovascular ingrowth and an outer layer of silastic that serves as a vapour and bacterial barrier.[12] It has been used to give a good effect in clean superficial burns and in donor sites. When used to cover partial-thickness wounds, the mesh adheres to the wound until healing occurs beneath. Biobrane® should be removed from any full-thickness wound prior to skin grafting.

Biobrane® is an established synthetic dressing for burn wounds, particularly in the paediatric population. Whitaker *et al*. published a critical evaluation of the evidence base for the varied uses of Biobrane® within the field of plastic and reconstructive surgery.[13] They concluded that there is good evidence (Grade A) to support the use of Biobrane® in the management of burns, particularly in partial-thickness burns in children. When dressed with Biobrane®, patients with superficial partial-thickness burns experience less pain as compared to gauze and silver sulfadiazine dressing.[14] Biobrane® also significantly reduces hospital stay, wound healing time and requirements of pain medications.[15] There are reported applications in patients with toxic epidermal necrosis, chronic wounds, or following skin resurfacing.[13]

However, Biobrane® has been associated with permanent scarring in partial-thickness scald wounds.[16] In general, Biobrane® has been shown to possess a wealth of potential uses. Further prospective clinical trials are warranted if these new applications are to become more widely accepted.

### Dermagraft®

Dermagraft® (Advanced BioHealing, LaJolla, CA, USA) is a bioabsorbable polyglactin mesh seeded with allogenic neonatal fibroblast.[17] Indications for the usage of Dermagraft are in burn wounds, chronic wounds and diabetic ulcers.[8] It can be used as a temporary or permanent covering to support the take of meshed split-thickness skin grafts on excised burn wounds.[17,18]

Dermagraft® appears to produce results as good as allograft with regard to wound infection, wound exudate, wound healing time, wound closure and graft take. It was also reported to be removed easier than allograft, with significantly higher level of patient satisfaction.[8,19] There has been no adverse reactions to Dermagraft®, with no evidence of rejection, early deterioration or separation from wound.[18] There has so far been no safety issues regarding Dermagraft®.[19]

### Integra®

Integra® (Integra LifeSciences Corp., Plainsboro, NJ, USA) is a dermal regeneration template consisting of bovine collagen, chondroitin-6-sulphate and a silastic membrane. This product has gained widespread use in the clinical treatment of deep partial-thickness and full-thickness burn wounds, full-thickness skin defects of different aetiologies, chronic wounds and in soft tissue defects.[8,17,20,21] The bovine collagen dermal analogue integrates with the patient’s own cells and the temporary epidermal silicone is peeled away as the dermis regenerates. A very thin autograft is then grafted onto the neo-dermis.[17,22] Heimbach *et al*. showed that Integra® was superior to autograft, allograft or xenograft in terms of wound healing time.[21] However, with regard to wound infection and graft take, Integra® did not produce as good a result.[21,23]

### Apligaft®

Apligraft® (Organogenesis, Inc., Canton, MA, USA, and Novartis Pharmaceuticals Corp., East Hanover, NJ, USA) is a bilayered living skin equivalent. It is composed of type I bovine collagen and allogenic keratinocyte and neonatal fibroblast.[17,22] It is indicated in partial to full thickness burns, skin graft donor sites, chronic wounds, diabetic ulcers and Epidermolysis Bullosa.[8] It has to be applied “fresh” as it has a shelf-life of 5 days at room temperature.[17] Apligraft® has been shown to accelerate wound closure. Apligraft® when combined with autograft has produced more favourable results than autograft only. Scar tissue, pigmentation, pliability and smoothness were significantly closer to normal with Apligraft®.[24]

### Matriderm®

Matriderm® (Skin and Health Care AG, Billerbeck, Germany) is a structurally intact matrix of bovine type I collagen with elastin. It is utilised for dermal regeneration. Its indications are full thickness or deep dermal burn wounds and chronic wounds. The matrix serves as a support structure for the ingrowth of cells and vessels. Its elastin component improves the stability and elasticity of the regenerating tissue. As the healing process advances, fibroblast lays down the extracellular matrix and the Matriderm® resorbes.[25] Its indications seem to be similar to Integra®. Schneider *et al*. compared the engraftment rate and rate of vascularisation of Matriderm® and Integra® in a rat model. They revealed no major differences in engraftment rates or vascularisation.[26]

However, unlike Integra, Matriderm has been shown to be able to accommodate immediate split thickness skin grafting with no diminished take.[27] In experimental models, the matrix reduces wound contracture, and histologically collagen bundles in the scar are more randomly orientated. Clinical trials with a long-term clinical evaluation showed no difference in scar elasticity between the described dermal substitute and split thickness grafts alone.[27] However, there is still lack of clinical data on the development of wound contracture.

### OrCel®

OrCel® (Fortificell Bioscience, NY, USA) is a bilayered cellular matrix in which normal human allogeneic skin cells (epidermal keratinocytes and dermal fibroblasts) are cultured in two separate layers into a type I bovine collagen sponge. OrCel® is a bilayer dressing resembling normal skin and was developed as a tissue-engineered biological dressing. It is indicated in the treatment of chronic wounds and skin graft donor sites. OrCel® has also been used as an overlay dressing on split-thickness skin grafts to improve function and cosmesis.[8,28,29]

### Hyalomatrix®

Hyalomatrix® (Fidia Advanced Biopolymers, Padua, Italy) is a bilayer hyaluronan base scaffold with autologous fibroblast. It has an outer silicone membrane. The scaffold delivers hyaluronan to the wound bed, and the silicone membrane acts as a temporary epidermal barrier.[30] It is indicated in burn wounds and chronic wounds.[8]

## CONCLUSION

Skin substitutes have important roles in the treatment of deep dermal and full thickness wounds of various aetiologies. At present, there is no ideal substitute in the market that provides an effective and scar-free wound healing. Further research should be carried out not only to compare different skin substitutes but also to evaluate new biological and synthetic materials that can be utilised in wound healing.
